# StockData: An open investment transaction dataset

**DOI:** 10.1016/j.dib.2026.112504

**Published:** 2026-01-27

**Authors:** Mingrui Li, Can Wu, Wentao Mao, Elif E. Firat, Robert S. Laramee

**Affiliations:** a Orange Digital Technology Co., Ltd., China; b The School of Computer Science, University of Nottingham, United Kingdom; c Shanghai Foreta Technology Co., Ltd., China; d Computer Engineering Department, Cukurova University, Turkey

**Keywords:** Financial data, Stock market, Data visualization

## Abstract

As the number of investment transactions grows, so does the importance of visual analysis to study financial data. Despite modern stock market platforms and research tools offering a range of stock data and visual analysis software, retail investment data is difficult to find due to privacy and security concerns. This challenge poses barriers to researchers and analysts interested in portfolio management, analysis, and visualization. This paper introduces StockVis, the first open and anonymized dataset of investment transactions from an individual investor. This freely accessible dataset can be used to study investment portfolio analysis, thereby improving strategic decision-making in portfolio management. StockVis features a comprehensive set of investment transactions focused on the U.S. stock market, encompassing the transaction records of a single anonymous investor over 3–4 years, complemented by derived metadata on the stocks of interest. We provide an overview of the dataset, detailing its features and the anonymization process and present some case studies and illustrative exemplar images as a foundation for further study. We are confident that the accessibility of this open data will significantly contribute to the research community, fostering enhanced exploration in the field of investment.

Specifications Table

**Table:** 

Subject	Social Sciences
Specific subject area	Investment Transaction Data
Type of data	Table, Raw, Analyzed, Processed
Data collection	The first open, anonymized investment transaction data set from a retail investor. The open data set features over 2600 investment transactions, such as buys, sells, and dividends from individual public companies, spanning a time period of 3–4 years.
Data source location	*School of Computer Science, University of Nottingham, UK*
Data accessibility	Repository name: Mendeley
	DOI: 10.17632/k4tnk2nggv.1
	URL: https://data.mendeley.com/datasets/k4tnk2nggv/1
Related research article	URL: https://doi.org/10.2312/evs.20231052

## 1 Value of Data

As the volume of investment transactions is constantly increasing (over a billion transactions per day [1]), so does the importance of visual analysis to support financial data analysis. Despite the growing popularity and number of platforms offering statistical and graphical analysis of finance data, there is a lack of accessible data on individual retail investment transactions. This is due to privacy and security reasons. Analysis of investment transaction data can facilitate informed decisions with respect to behavior, investing, or risk management. It can also assist in tracking and detecting investing trends, the psychology of investing, and other retail financial activities. However, the data we present here must be interpreted with caution as it represents one investor; therefore, generalizations may not follow to general investing patterns.

There are other finance-related datasets online that can be used for research and analysis; however, there are often special access privileges or monetary fees posing barriers to their access [2]. Furthermore, we are unable to find an open dataset specifically of retail investment transactions. The datasets we find are usually associated with corporations, aggregate transactions, or financial statements, in other words, summaries of activity and decisions rather than individual transactions. Real datasets containing investment data are often hidden from the public for a variety of reasons, including data privacy and security concerns from both retail investors and retail trading platforms.

In this paper, we present the first open, anonymized investment transaction data set from a single retail investor. The open data set features over 2600 investment transactions, such as buys, sells, and dividends from individual public companies, spanning a time period of 3–4 years. It offers a rich set of financial activities that can be used to study the financial behavior of an individual, derive case studies for visual analysis, and beyond. This paper contributes the following:

• The first anonymized, open, investment transaction data set for visualization and beyond
• A thorough description of the data, as well as a set of metadata that can be used to study an investor’s decision-making
• The first set of initial images of the investment transaction data

However, we emphasize the individual nature of the dataset, and therefore any generalizations must be made with caution and supported with further evidence.

**Classic Datasets:**The graphics and visualization literature features datasets that have become classics, i.e., used as exemplars in hundreds of research papers. The first classic data set comes from computer graphics, namely, the teapot [3]. Another classic data set is the Stanford Bunny [4] originally published by Turk and Levoy [5]. The teapot and Stanford Bunny datasets are used as standard benchmarks for many rendering algorithms. A classic data set that is used throughout the flow visualization literature is the tornado [6] released by Roger Crawfis. In addition, the Iris [7] and Cars[8] data sets are featured in many parallel coordinate plots in the literature [9]. Last year, retail bank transactions data were released [2]. We hope the dataset we introduce here evolves into a classic investment transactions benchmark.

## 2 Background

**Overviews and Surveys:** McNabb and Laramee [10] introduce a unique classification system of information visualization survey papers that offers an overview of the related work. A survey conducted by Shi et al. [11] presents a comprehensive examination of research that visually examines abnormal user behavior and classifies it within the financial transaction area, related to the movement of money when buying and selling. The survey by Roberts and Laramee [12] explores trends in business data visualization, segmenting literature into business intelligence and customer-centric data. Their literature classification spans areas such as business intelligence, the study of business ecosystems, and the analysis of data centered on consumers. Another survey by Ko et al. [13] compiles research on stock trading and fund management, emphasizing traditional 2D visualization methods and innovative techniques like interactive Kohonen maps by Schreck et al. [14] for grouped trajectory data analysis.

**Visualization of Investment Transactions:**Sorenson and Brath [15] present a novel visualization method that merges continuous time-series data with discrete events in financial markets. Their approach incorporates interactive features to enhance the comprehension of financial trends and news impacts, offering a dynamic perspective on financial analytics. Hua et al. [16] propose an innovative fusion of force-directed algorithms with time-series charts to analyze stock market structures and connections. Their methodology emphasizes a detailed analysis of interconnected stock relationships, employing a mix of graphical and time-series images to unearth complex patterns within financial data. Chang et al. [17] present WireVis, a multiview methodology that facilitates analysts in examining extensive categorical time-varying data, including wire transactions.

VisFAN, a software application developed by Didimo et al. [18], is introduced as crime detection by visualizing financial activity networks. The researchers combine advanced techniques for graph sketching with tools for social network analysis and automated report production to provide innovative algorithms and interfaces for visually analyzing interconnected datasets. The paper by Singh and Best [19] examines the effectiveness of visualization tools for detecting money laundering patterns, aiming to prevent financial crimes.

The paper by Arleo et al. [20] examines the difficulties associated with modelling financial dynamics. Sabrina 2.0 is introduced as a visual analytics approach that facilitates examining financial data at various dimensions and produces networks of firm-to-firm financial transactions to offer insight into the economic climate. Leite et al. [21] presents EVA, a system that employs a visual analytics method to detect financial fraud. Subsequently, Leite et al. [22] introduce NEVA, a system designed to identify and analyze illicit networks involved in bank transaction events. The approach also facilitates investigating intricate relationships and interconnections within the data. Rudolph et al. [23] introduces FinVis, a visual analytics tool designed to assist non-expert users in understanding the correlation elements of financial data and making informed personal finance decisions.

**Open Finance Data:** Firat et al. [2] introduce *MoneyVis*, the first open data set of anonymized bank transactions. This data is useful for uncovering complex consumer spending patterns and providing a deeper understanding. This paper extends the scope of MoneyVis and provides the first comprehensive open data set of investment transactions concentrated on the U.S. stock market. The paper presents initial analyses and graphs to serve as a foundation for future exploration in the field of investment, contributing a valuable starting point for guiding investors’ decision-making and beyond.

## 3 Data Description

This section describes the data anonymization process, the raw brokerage transaction records, and the metadata spreadsheet we created to facilitate the evaluation of portfolio performance.

### 3.1 Description of transactions

The raw investment data consists of over 2700 online brokerage transaction records over three years spanning 2020–2024.

**Anonymization**: We have taken steps to anonymize the data, including removing all identifier information such as name and original account numbers. Without an associated name or ID, it is not possible to identify any individual retail investor since there are billions of stock market transactions every day. Each transaction features the following fields:

1. Action: describes the type of transaction that occurred:
  • Market Buy,
  • Market Sell,
  • Dividend: There are seven different types of dividends: bonus, demerger, dividends paid by foreign corporations, dividends paid by US corporations, ordinary manufactured payment, ordinary return of capital non-US, interest on cash,
  • Deposit,
  • Withdrawal,
2. Time: the time of the transaction,
3. ISIN: International Securities Identification Number, a unique code that identifies a globally tradable security,
4. Ticker: the stock ticker symbol, which is a code used to uniquely identify a specific stock across trading platforms,
5. Name: the name of the stock, typically the name of a company,
6. No. of shares: the number of shares involved in the transaction,
7. Price/share: the transaction price per share
8. Currency: the currency of the transaction
9. Exchange rate: the exchange rate at the time of the transaction,
10. Result (GBP): the profit or loss result when selling stocks, in British pounds
11. Total (GBP): the total transaction amount in British pounds.

The transaction records contain 10 additional fields providing data on tax fees, transaction IDs, and additional notes. A description of these is provided in the dataset itself.

### 3.2 Description of metadata

In addition to the raw data transaction records, we created a spreadsheet of company-centric metadata to facilitate the overall performance of the portfolio. (with transactions until 12 Dec 2023) Each record features the following attributes:

1. Row Number: A sequential identifier for each transaction entry.
2. Company Name: The company’s full legal name involved in the transaction.
3. Ticker Symbol: The stock market symbol representing the company’s publicly traded shares.
4. Additional company and transaction metadata symbols and their meanings are provided in Table 1.

**Table 1:** Stock Transaction Symbols and their meanings. Uppercase symbols denote total amounts, whereas lowercase symbols denote individual transactions.This is a summary of the company metadata provided in the Google Sheet.

Symbol	Description

$S_{pur}$	Total Number of Shares Purchased: The sum of all shares bought across different transactions.
$P_{tot}$	Total Purchase Amount: The cumulative monetary value of all shares purchased.
$p_{avg}$	Average Purchase Price per Share, the Total Purchase Amount divided by the Total Number of Shares Purchased: $p{\left(c\right)}_{avg}=P{\left(c\right)}_{tot}/S{\left(c\right)}_{pur}$ where c indicates the company shares purchased.
$S_{sale}$	Total Number of Shares Sold.
$R_{tot}$	Total Sales Amount, The aggregate monetary value received from selling shares.
$r_{avg}$	Average Sale Price per Share: $r_{avg}=R_{tot}/n$, where n is the number of shares sold.
$S_{net}$	Net Total Number of Shares, the difference between the Total Number of Shares Purchased and the Total Number of Shares Sold, $S_{net}=S_{pur}-S_{sale}$
$p_{cur}$	Current Share Price, the latest trading price of the shares indicated by Google Finance. $p{\left(c\right)}_{cur}$ indicates the current share price for company c
$D_{tot}$	Total Dividends: The total dividends received by the specified date.
CGR	Realized Capital Gain & Loss: The profit or loss realized from selling shares. $CGR\left(c\right)=(p{\left(c\right)}_{sale}-p{\left(c\right)}_{avg})\cdot n$, where c is the company ticker symbol, and $p{\left(c\right)}_{sale}$ indicates the sale price of the shares for company, c.
CGU	Unrealized Capital Gain & Loss: The potential profit or loss not yet realized on shares still held. $CGU\left(c\right)=(p{\left(c\right)}_{cur}-p{\left(c\right)}_{avg})\cdot n$

The dataset is live in the sense that it stays up-to-date by using the Google Finance API to fetch the current price of all stocks featured in the dataset. Thus, the analysis of good versus bad decisions can change over time as a company’s stock prices evolve. This is one reason we mention *the date* each image is created in our case studies in the next section. Each image changes over time. Note that there are additional fields in the dataset that we do not describe here for conciseness, e.g., the fees associated with converting currencies, which can influence ROI due to the fluctuation of currency exchange rates. This is an interesting direction for future work.

### 3.3 Data access

The following URL provides read access to the open Investment transaction data set:

https://data.mendeley.com/datasets/k4tnk2nggv/1

## 4 Experimental Design, Materials and Methods

**Fig. 1:**
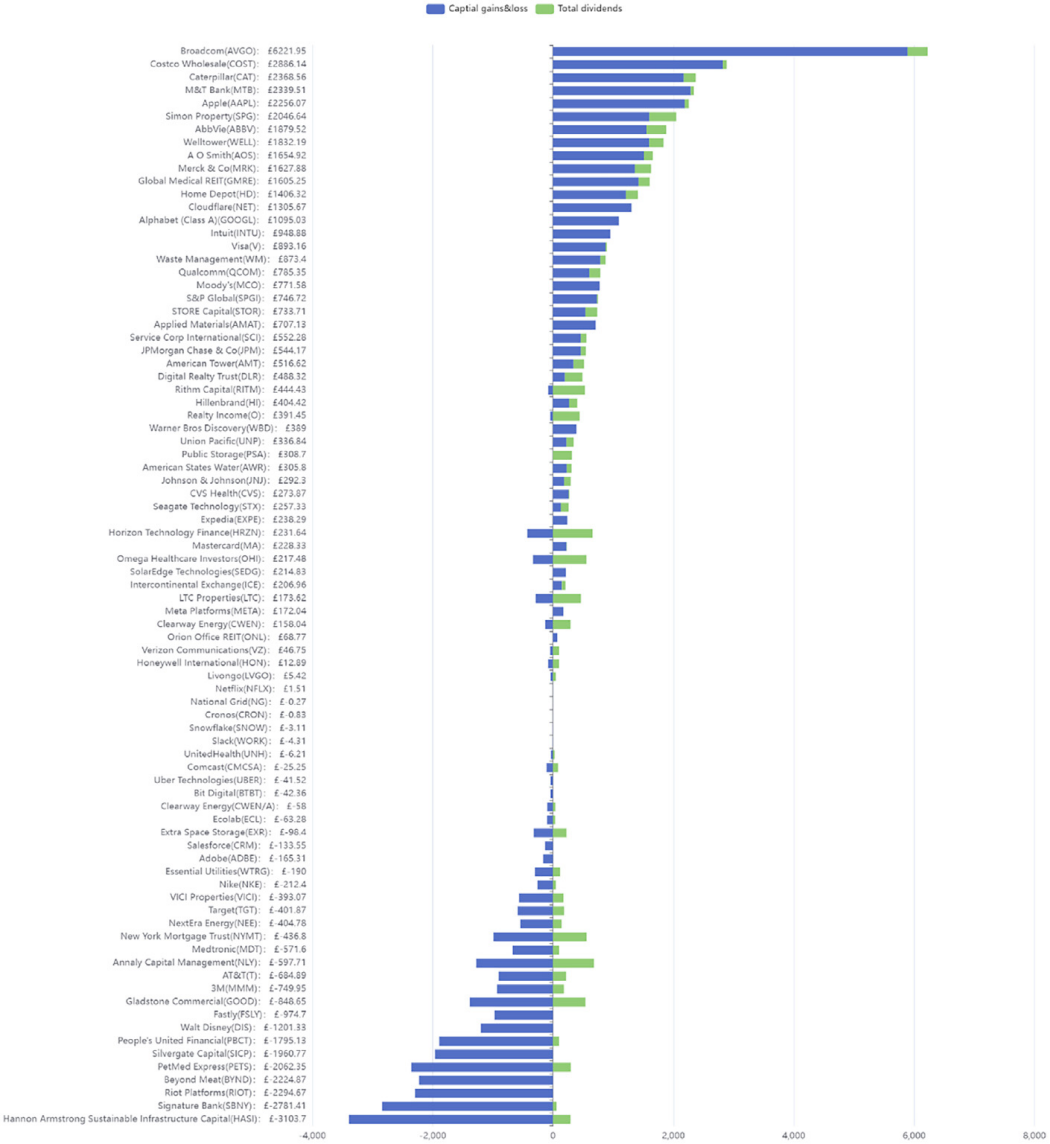
Stacked Bar Chart. This image shows an illustrative example of 83 equities reveals the extremes of investment performance as of 27 December 2023: Broadcom (AVGO) exhibits the highest return of £6,221.95, whereas Hannon Armstrong Sustainable Infrastructure Capital (HASI) demonstrates the lowest return of -£3,103.7.

This section uses the investment data to generate detailed images. These graphics offer illustrative examples of investment activities. We provide a supplementary video with further illustrative example of the data:

Example 1 Overview Stacked Bar Chart: Fig. 1 (see Fig. 1 for a high-resolution version) presents a stacked bar chart designed to reflect company-centric metadata. The chart compiles investment data from April 2020 to December 2023 and depicts the cumulative returns on different equities, encompassing dividend income and capital gains. This imagery clearly shows the variation in returns among different equities. As an example, Broadcom (AVGO) has the best return of £6,221.95, while Hannon Armstrong Sustainable Infrastructure Capital (HASI) has the lowest return of -£3,103.70. Costco Wholesale (COST) showed higher performance with £2,886.14, Caterpillar (CAT) at £2,368.56, and M&T Bank (MTB) at £2,339.51. The remaining businesses in the top ten are Apple (AAPL) with a value of £2,256.07, Simon Property (SPG) at £2,046.64, AbbVie (ABBV) with £1,879.52, Welltower (WELL) at £1.832.19, A O Smith (AOS) with £1,654.92, and Merck&Co (MRK) with £1,627.88.

The chart analyses the relationship between investment returns, capital gains, and dividends. Illustratively, Rithm Capital (RITM) incurred a capital gains loss of -£83.14 and a total return of £444.43 due to dividends amounting to £527.57. The Rithm Capital (RITM) analysis highlights a situation where high dividend returns offset capital gains losses. Although the dividends are substantial, this scenario reveals the significant associated investment risks, underscoring the need to reevaluate the investment value carefully.

**Fig. 2:**
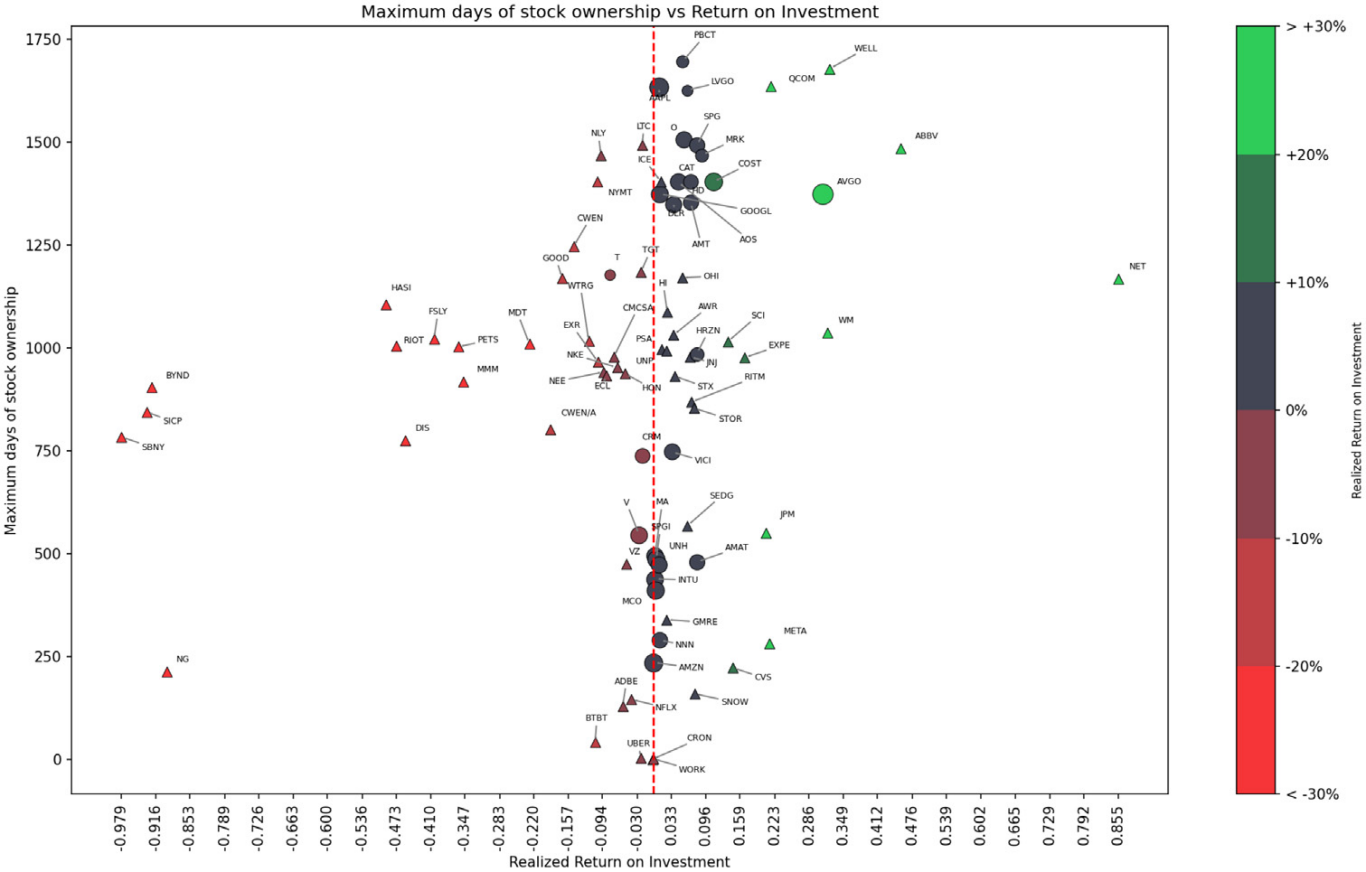
Realized ROI Scatterplot created with Matplotlib and adjustText on February 25, 2025. Stock Holding Period vs. Realized ROI for 82 equities. Colormap encodes ROI magnitude as a percentage (red: negative, green: positive), with a dashed line at ROI=0%. (For interpretation of the references to colour in this figure legend, the reader is referred to the web version of this article.)

**Fig. 3:**
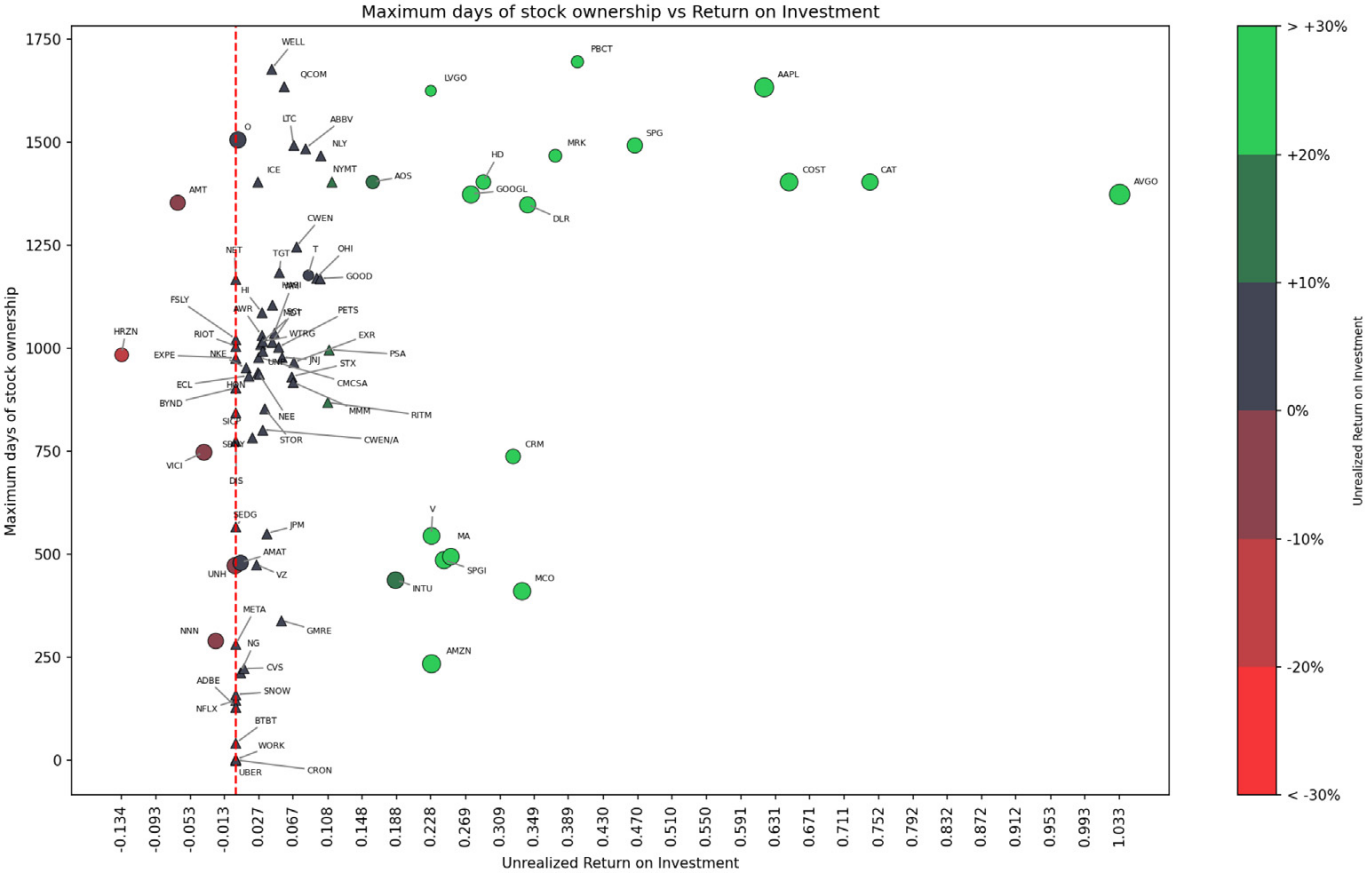
Unrealized ROI Scatterplot created with Matplotlib and adjustText on February 25, 2025. Stock Holding Period vs. Unrealized ROI for 82 equities showing potential returns on current holdings. Note the more polarized distribution compared to realized returns.

Example 2 ROI Scatterplot: Figs. 2 and 3 present two scatterplots that are based on each company’s stock performance and company metadata. The charts summarize investment data from April 2020 to July 2024 and describe the relationship between the ROI of different stocks and the maximum number of days held. The glyth color, size, and shape show the degree of ROI, the current shareholding amount, and whether the stock is currently held, respectively. Specifically, triangles indicate sold holdings, while circles represent active holdings, with glyph size proportional to current investment. In addition, a red dotted line indicates the scale line where the ROI is 0. For example, Broadcom (AVGO), which, at that time, was the largest holding, has the highest ROI of 0.976, and its maximum holding period is about 1400 days; while Signature Bank (SBNY), which has been sold, has the lowest ROI of -0.979, and its maximum holding period is about 800 days.

*Realized ROI.* In the following, Realized ROI is calculated using the formula:

$$\begin{array}{cc} \text{Realized}\;\text{ROI(c)} & =\frac{CGR\left(c\right)+D{\left(c\right)}_{tot}}{P{\left(c\right)}_{tot}}\\ & =\frac{R{\left(c\right)}_{tot}-P{\left(c\right)}_{tot}+D{\left(c\right)}_{tot}}{P{\left(c\right)}_{tot}}\\ & =\frac{\left(p{\left(c\right)}_{sale}-p{\left(c\right)}_{avg}\right)\cdot n+D{\left(c\right)}_{tot}}{p{\left(c\right)}_{avg}\cdot n} \end{array}$$

where, c, is the company sold and n is the number of shares.

*Unrealized ROI.* Similarly, the formula for the Unrealized ROI can be presented as:

$$\begin{array}{cc} \text{Unrealized}\;\text{ROI} & =\frac{CGU\left(c\right)+D{\left(c\right)}_{tot}}{P{\left(c\right)}_{tot}}\\ & =\frac{\left(p{\left(c\right)}_{cur}-p{\left(c\right)}_{avg}\right)\cdot n+D{\left(c\right)}_{tot}}{p{\left(c\right)}_{avg}\cdot n} \end{array}$$

In this case, we treat dividends from realized and unrealized stock the same. These might be theoretically separable for other types of analysis. For our purposes we treat them the same to reduce unnecessary complexity.

**Fig. 4:**
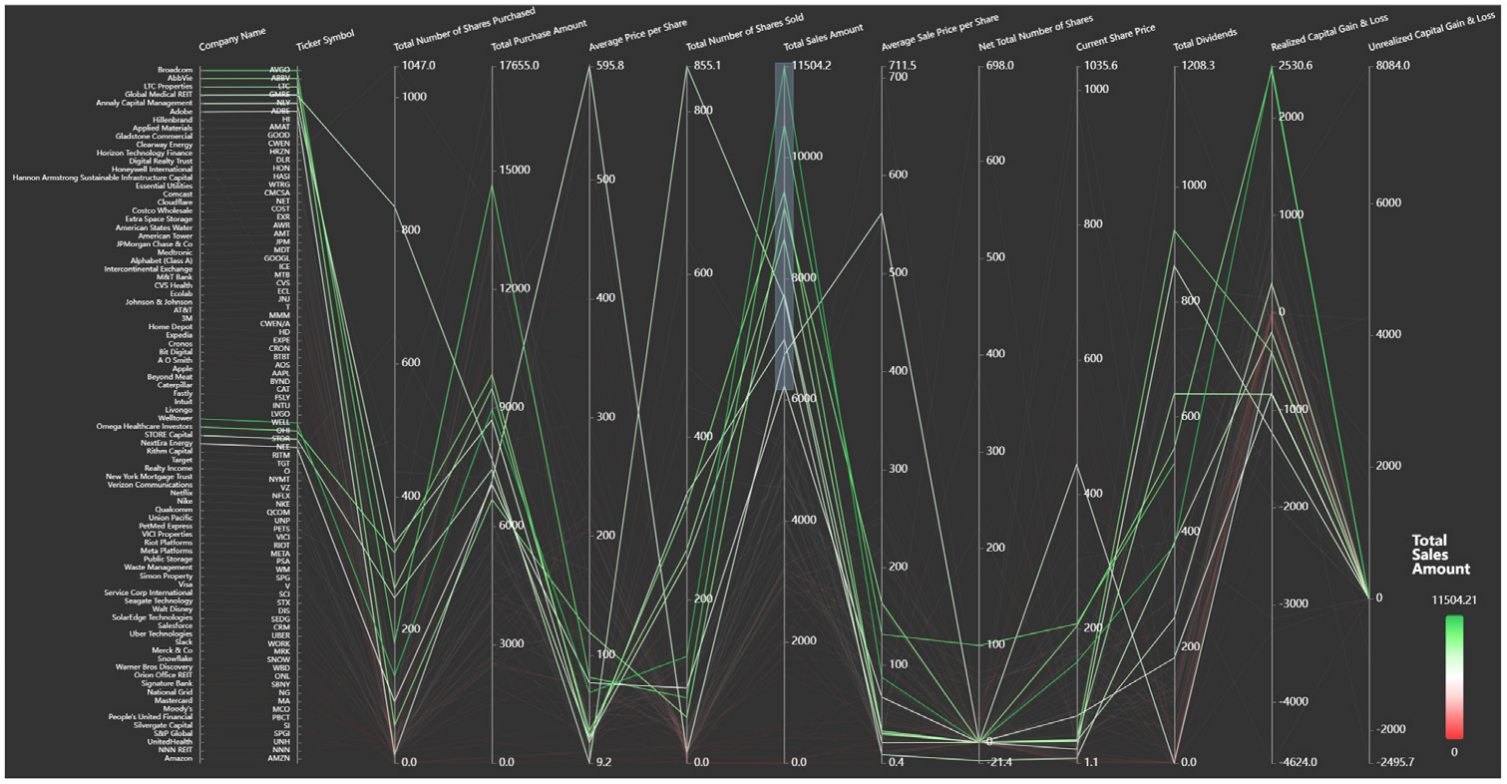
A parallel coordinate plot created with ECharts on February 25, 2025, that visualizes company investment portfolio metrics across multiple dimensions. The visualization highlights the top 10 companies based on Total Sales Amount, with green indicating higher stock sale values. (For interpretation of the references to colour in this figure legend, the reader is referred to the web version of this article.)

Example 3 Company Metadata: Parallel Coordinates: Parallel coordinate plots are a powerful technique for effectively visualizing and understanding multidimensional data [24]. When applied to investment data, this design can display high-dimensional transactions with up to 10–15 dimensions, since each axis is visually separated [25]. Fig. 4 represents each company’s metadata in the dataset, including various company metrics across multiple attributes, enabling comparative analysis. The design includes dimensions: company name, total shares purchased, purchased amount, total number of shares sold, average sale price per share, total sales amount, realized capital gains and losses, and more. In this plot, only stock sales with a total sales amount exceeding $6,000 are displayed (brushing). The highest stock sales are highlighted in green. If we turn off brushing, the lowest total stock sales amount is marked in red. This approach facilitates comparison between company data variables and offers valuable insight into how decisions are made with respect to stock purchases and sales, supporting informed investment decisions.

**Fig. 5:**
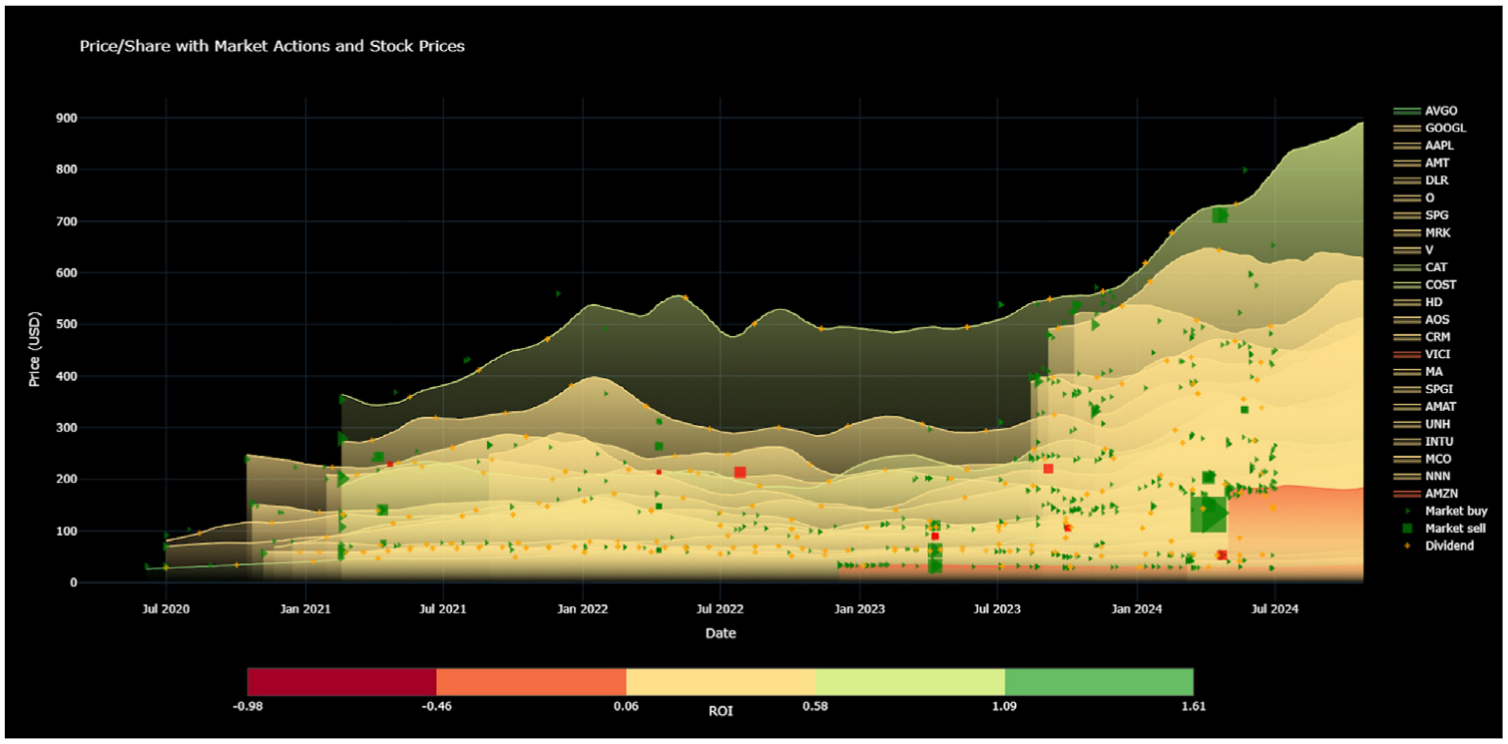
Comparison Line Graph created with plotly, on 27 October, 2024: This image shows the stocks with positive ROI that are still held. The lines depict the 50-day moving average prices of each stock. The color of each curve represents the ROI over time. The triangle glyphs represent market buys, the rectangle glyphs represent market sells. The red rectangles represent sells at a loss, the green glyphs represent sells at a profit. The yellow glyphs represent dividends. The size of each glyph represents the amount of the trade. (For interpretation of the references to colour in this figure legend, the reader is referred to the web version of this article.)

Comparison Line Graph: Fig. 5 illustrates multiple lines representing the 50-day moving average price of the stocks with positive ROI in the portfolio. There are 23 stocks with positive ROI at the time this image was generated. There are 4 types of glyphs in the chart. Dividends are represented by small yellow cross shapes. Market buys are indicated using green triangles. Profitable market sales, whose sale price is higher than or equal to the average purchase price per share, are visualized as green squares. Unprofitable market sales, whose current price is lower than the average purchase price per share, are represented by red squares. The size of the glyphs is proportional to their amount of each trade. As Fig. 5 shows, there are only a few unprofitable sales in the illustrated stocks.

## 5 Limitations

The data can be used for various purposes and will be very helpful for those interested in the combination of visualization and finance. However, we believe it will also be very useful to analysts outside the visualization community, such as in business or finance. We provide a description of the raw transaction data as well as a supplementary spreadsheet of metadata that can be used for portfolio analysis. Limitations include:

• Visualization: a lack of more advanced and novel visual designs of the investment transaction data and the performance metadata.
• Time: The original set of transactions is limited to a four year time span. However, this is partially mitigated by using the Google API to retreive current stock prices.
• Metadata: the dataset itself could also be enhanced with new or updated metadata.
• The dataset represents only one individual investor, therefore caution must be used if attempting to make any generalization from its use.

## CRediT Author Statement

ML, CW, and WM contributed to data pre-processing, writing the manuscript, and creating the visualizations. EEF contributed to writing the manuscript and the visualization design. RSL presented the main idea, contributed to data collection, wrote and proofread the manuscript, and supervised the entire project. All authors read and approved the final manuscript.

## Ethics Statement

The authors have read and follow the ethical requirements for publication in Data in Brief and confirm that the current work does not involve human subjects, animal experiments, or any data collected from social media platforms.

## Data Availability

Stock Transactions DatasetStock Transactions Dataset (Original data).

## Appendix B Supplementary materials

Supplementary Data S1 Supplementary Raw Research Data. This is open data under the CC BY license http://creativecommons.org/licenses/by/4.0/ Supplementary Data S1
